# A systematic review and meta-analysis demonstrating Klotho as an emerging exerkine

**DOI:** 10.1038/s41598-022-22123-1

**Published:** 2022-10-20

**Authors:** Hugo de Luca Corrêa, Artur Temizio Oppelt Raab, Thamires Marra Araújo, Lysleine Alves Deus, Andrea Lucena Reis, Fernando Sousa Honorato, Paolo Lucas Rodrigues-Silva, Rodrigo Vanerson Passos Neves, Henver Simionato Brunetta, Marcelo Alves da Silva Mori, Octávio Luiz Franco, Thiago dos Santos Rosa

**Affiliations:** 1 Graduate Program of Physical Education, Catholic University of Brasilia (UCB), EPTC, QS07, LT1 S/N, Bloco G Sala 119, Águas Claras, Taguatinga, Brasília, Distrito Federal CEP 72030-170 Brazil; 2 Faculty of Medicine, Catholic University of Brasilia, Brasília, Distrito Federal Brazil; 3 Faculty of Bio-Medicine, Catholic University of Brasilia, Brasília, Distrito Federal Brazil; 4 Embrapa Genetic Resources and Biotechnology, Brasilia, Distrito Federal 70000-000 Brazil; 5 Department of Biochemistry and Tissue Biology, University of Campinas, Campinas, Brazil; 6 Obesity and Comorbidities Research Center, University of Campinas, Campinas, Brazil; 7 Experimental Medicine Research Cluster, University of Campinas, Campinas, Brazil; 8 Centro de Análises Proteômicas e Bioquímicas, Pós-Graduação em Ciências Genômicas e Biotecnologia, Universidade Católica de Brasília, Brasília, DF Brazil; 9 S-Inova Biotech, Pós-Graduação em Biotecnologia, Universidade Católica Dom Bosco, Campo Grande, MS Brazil

**Keywords:** Molecular biology, Biomarkers, Endocrinology, Molecular medicine

## Abstract

Klotho is an anti-aging protein with several therapeutic roles in the pathophysiology of different organs, such as the skeletal muscle and kidneys. Available evidence suggests that exercise increases Klotho levels, regardless of the condition or intervention, shedding some light on this anti-aging protein as an emergent and promising exerkine. Development of a systematic review and meta-analysis in order to verify the role of different exercise training protocols on the levels of circulating soluble Klotho (S-Klotho) protein. A systematic search of the Cochrane Central Register of Controlled Trials (CENTRAL), MEDLINE through PubMed, EMBASE, CINAHL, CT.gov, and PEDro. Randomized and quasi-randomized controlled trials that investigated effects of exercise training on S-Klotho levels. We included 12 reports in the analysis, comprising 621 participants with age ranging from 30 to 65 years old. Klotho concentration increased significantly after chronic exercise training (minimum of 12 weeks) (Hedge’ *g* [95%CI] 1.3 [0.69–1.90]; *P* < 0.0001). Moreover, exercise training increases S-Klotho values regardless of the health condition of the individual or the exercise intervention, with the exception of combined aerobic + resistance training. Furthermore, protocol duration and volume seem to influence S-Klotho concentration, since the effect of the meta-analysis changes when subgrouping these variables. Altogether, circulating S-Klotho protein is altered after chronic exercise training and it might be considered an exerkine. However, this effect may be influenced by different training configurations, including protocol duration, volume, and intensity.

## Introduction

In Greek mythology, even the most powerful gods needed to bow to the three Moirai: Clotho, Lachesis, and Atropos. These three goddesses of fate personified the inescapable destiny of mortals by controlling the thread of life^1^. Clotho spun the thread of life, Lachesis measured and allotted it to each person, and Atropos was the thread cutter. In view of the Moirai story, when Kuro-o et al. discovered the gene that seems to control aging, they named it Klotho^2^.

Klotho is now considered a strong biomarker and therapy for several diseases^3–10^. In addition, it presents anti-inflammatory and antioxidant properties^11–13^. Klotho increases the affinity of fibroblast growth factors (FGFs) 19, 21, and 23 for their respective receptors^14^. The FGF23-Klotho axis acts by suppressing renal inorganic phosphate reabsorption and activating vitamin D biosynthesis^14^. This pathway prevents calciprotein particle formation and, consequently, reduces cardio-renal damage^14,15^. Furthermore, the inflammation and cardiac dysfunction provoked by aging are associated with partial Klotho deficiency^16^. Part of this mechanism is proposed to be due to Klotho’s capacity to regulate energy metabolism and induce the expression of antioxidant enzymes, including catalase and superoxide dismutase^17,18^.

Klotho supplementation improves blood pressure and renal parameters in a pre-clinical model of type 2 diabetes^15,19^. It also suppresses inflammation and improves cardiac function in aged, endotoxemic mice^16^. Zhang et al.^20^ demonstrated that the peroxisome-activated receptor-γ (PPAR-γ) increases Klotho expression, while the PPAR-γ antagonist inhibits Klotho in mouse kidney. Interestingly, exercise training promotes PPAR-γ activation in skeletal muscle and other tissues^21–23^, which might be linked to greater circulating levels of Klotho in different populations.

Exercise training induces Klotho secretion by skeletal muscle^24^, along with other molecules including irisin^25^, sestrin-2^26^, and a myriad of microRNAs^27^. The molecules produced and secreted by tissues and organs as a direct or indirect consequence of exercise are called exerkines^28,29^. Due to their therapeutic potential as exercise mimetics, exerkines open a novel avenue for drug discovery^28–31^. Although Klotho has promising therapeutic potential in human pathology, it has never been considered an exerkine until now. Therefore, knowing what the effects of exercise are on S-Klotho expression and secretion may help scientists and coaches to prescribe the best protocols to increase the levels of this protein.

In the past few years, the interest of the scientific community and industry in the possibility of mimicking or potentializing the effects of exercise has increased considerably^32,33^. In this context, exerkines have been pursued as alternative treatments or strategies to improve health and/or performance^28,29^. Although it is an intriguing idea, there is still a long way to go before the creation of an exercise “pill or shot”. Therefore, it is worth investigating the possible bioactive compounds that should be targeted as novel therapies related to exercise mimetics. Indeed, some insights might appear in the study of Kurosu et al. who were able to attenuate aging-related senescence in mice with Klotho overexpression^34^. Thus, the increase in Klotho following exercise training may demonstrate a potential link between being physically active and delaying aging.

The aforementioned scenario may lead to a better training prescription aimed at anti-aging effects. Moreover, it could even generate a perspective for the use of Klotho measurement in the biological control of sports training and clinical rehabilitation programs. Here, we systematically described different exercise protocols capable of inducing Klotho levels. In that context, the purpose of the present study was to verify the role of different exercise training protocols on S-Klotho concentrations. The present review provides novel insights supporting exercise as a strong intervention to increase S-Klotho in humans. While this phenomenon appears to occur regardless of the condition (healthy or diseased) and training protocol (aerobic or resistance training), we outlined that the volume and duration of the intervention might play a significant role on S-Klotho changes.

## Results

### Participant characteristics

The PRISMA flow diagram of study selection is presented in Supplementary Fig. 1. From the 8 studies that met the inclusion criteria, 12 reports were included in the quantitative analysis. Amaro-Gahete et al.^35^ was split into 3 independent reports: (a) combined aerobic + resistance training; (b) only aerobic; (c) aerobic + electromyostimulation. Corrêa et al.^36^ was split into two independent reports: (a) conventional resistance training and (b) blood-flow restricted resistance training. Neves et al.^37^ was split into 2 independent reports: (a) conventional resistance training and (b) isometric resistance training.

There were five reports with healthy subjects and seven with diseased patients. The diseases analyzed were chronic obstructive pulmonary disease^38^, stage two of chronic kidney disease^36^, end-stage renal disease^37,39^, and coronary artery disease^40^. Moreover, five reports investigated the effect of resistance training^36–38^, five reports investigated aerobic training^35,40–42^, and only two studies examined the combined effect of aerobic and resistance training^35,39^. Publications ranged from 2014 to 2021. There was a total of 621 participants with age ranging from 30 to 65 years. According to the results of critical appraisal phase presented in supplementary Fig. 2, included studies are in low risk of biases.

### Exercise training characteristics

The exercise training characteristics are presented in Table 1. The interventions lasted from 12 to 24 weeks (17 ± 6 weeks), performed between 2 and 5 days per week. Studies that applied a resistance training protocol used percentages of the maximum force or 1RM test^36,38^ and perceived exertion scale^37^ to control the intensity of the protocol. The increase in S-Klotho protein in these studies ranged from 8.39 to 88.51%. It was notable that the authors also observed associated benefits such as improvement in functional performance, bone mineral density, inflammatory biomarkers, and attenuation of chronic kidney disease progression (when applicable).

**Table 1:** Population characteristics, exercise training details, and outcomes.

Author and date	Population		Intervention detail				Outcome	
	Age	Condition	Type	Weeks	Frequency (days/week)	Protocol	Klotho	Associated benefits
**Resistance training studies**								
Boeselt et al.^27^	60	COPD	Rehabilitation RT	12	2–3	35–75% maximal force	↑8.39%	Functional performance
Neves et al. (a)^26^	56.3	Hemodialysis patients	Conventional RT	24	3	OMNI scale 5–8	↑88.51%	Bone mineral biomarkers and bone mineral density
Neves et al. (b)^26^	56.3	Hemodialysis patients	Isometric RT	24	3	OMNI scale 5–8	↑18.18%	Bone mineral biomarkers
Corrêa et al. (a)^25^	58	Stage 2 CKD	Conventional RT	24	3	50–70% 1RM	↑20.1%	Blunts CKD progression
Corrêa et al. (b)^25^	58	Stage 2 CKD	BFR RT	24	3	30–50% 1RM	↑18.51%	Blunts CKD progression
**Aerobic training studies**								
Amaro-Gahete et al. (b)^24^	53.4	Sedentary middle-aged adults	HIIT	12	2	Session A: 95% VO_2max_; Session B:120% VO_2max_	↑34.06%	Association with better body composition
Amaro-Gahete et al. (c)^24^	53.4	Sedentary middle-aged adults	HIIT + whole body electromyostimulation	12	2	Session A: 95% VO_2max_; Session B:120% VO_2max_ with eletromyostimulation	↑55.81%	Association with better body composition
Matsubara et al.^29^	60	healthy and postmenopausal	Aerobic training	12	2–3	60–80% HR_max_	↑47.75%	Improved Arterial stiffness
Rahimi et al.^30^	30	Control: non athletes; Intervention: Athletes	Aerobic training	12	3	70–80% HR_max_	↑12.66%	Improved Pro-BNP
Saghiv et al.^28^	61	Coronary artery disease	Aerobic aquatic training	12	4–5	75–85% HR_max_	↑12.01%	Cardiac Hypertrophy indexes
**Combined training studies (aerobic + resistance training)**								
Amaro-Gahete et al. (a)^24^	53.4	Sedentary middle-aged adults	WHO recommendation of aerobic + RT	12	3	AT: 60–65% HR_res_; RT: 40–60% 1RM	↑47.75%	Association with better body composition
Fakhrpour et al.^31^	61	Hemodialysis patients	Aerobic + RT	16	3	AT: 45 min (12–14 Borg scale); RT: 40–65% 1RM	↑7.27%	Strength and functional performance

*COPD* chronic obstructive pulmonary disease, *RT* resistance training, *CKD* chronic kidney disease, *BFR* blood-flow restriction, *1RM *1-repetition maximum, *HIIT* high intensity interval training, *VO*_*2max*_ maximal oxygen uptake, *HR*_*max*_ maximal heart rate, *HR*_*res*_ reserve heart rate, *BNP* brain natriuretic peptide, *WHO* World Health Organization.

The studies that applied aerobic exercise used percentages of VO_2max_^35^ or HR_max_^40–42^ to control the intensity of the protocol. The increase in Klotho protein ranged from 12 to 55.8%. A positive association was verified with higher muscle mass, improvement in arterial stiffness, pro-brain natriuretic peptide, and cardiac hypertrophic indices. Only two of the included studies assessed the combined effect of aerobic + resistance training^35,39^. Amaro-Gahete et al.^35^ prescribed the protocol based on the World Health Organization recommendation (aerobic: 60–65% of the reserve heart rate; resistance: 40–60% of 1RM). Klotho increased by 47.75% and seemed to be associated with higher muscle mass. Fakhrpour et al.^39^ prescribed aerobic training based on the Borg scale (45 min; 12–14 on Borg scale) and resistance training based on 1RM (40–65% of 1RM). The authors found an increase of 7.27% in Klotho levels, concomitant to an increase in muscle strength and functional performance.

### Meta-analysis

**Figure 1:**
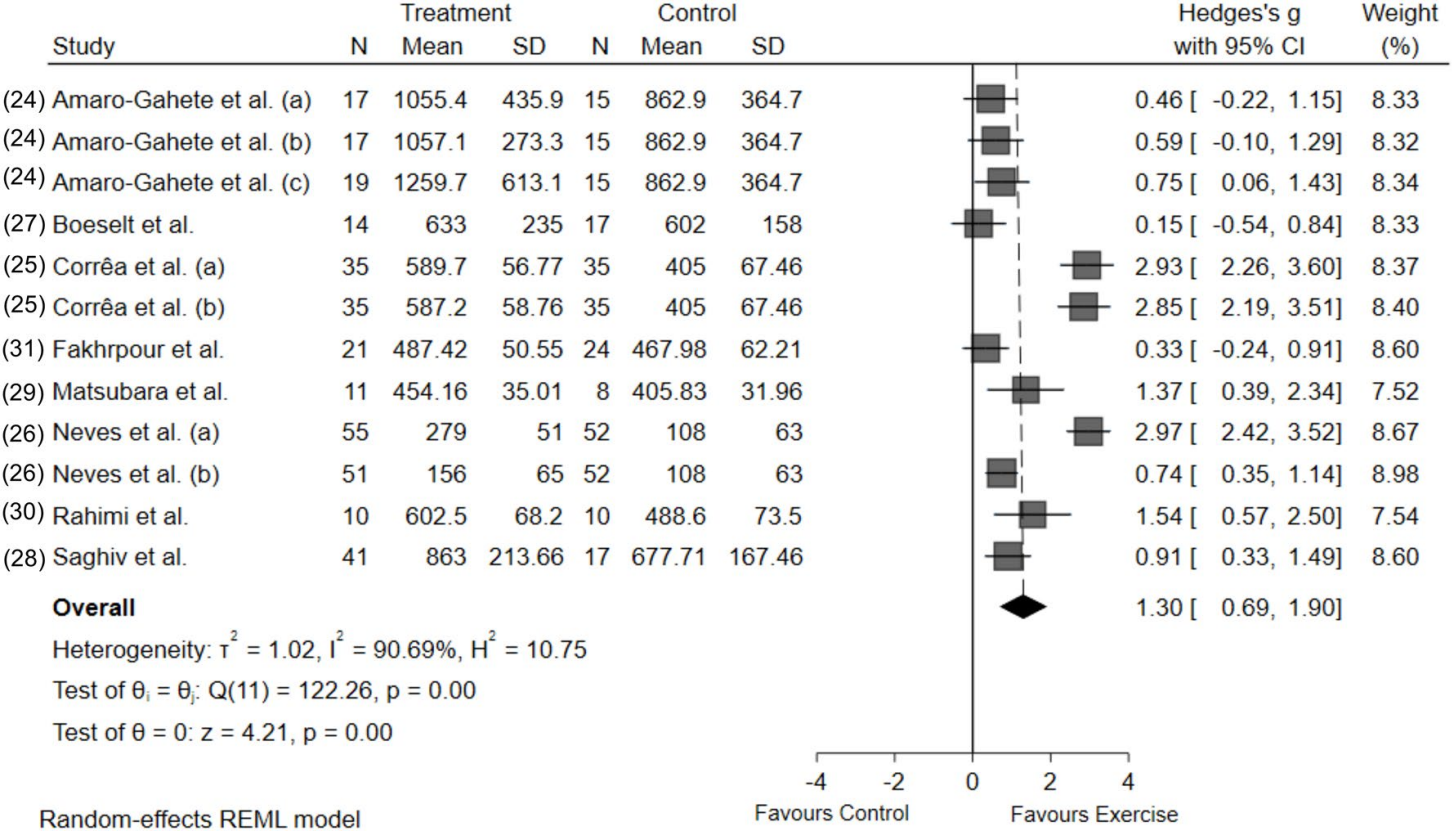
Forest plot of the results from a random-effects meta-analysis shown as Hedges’ g with 95% CIs on Klotho concentrations. For each study, the square represents the Hedges’ *g* of the intervention effect with the horizontal line intersecting it as the lower and upper limits of the 95% CI. The rhombi represent the total effect.

Figure 1 illustrates the overall response of Klotho protein to exercise training. Chronic exercise training increased circulating Klotho, independent of the modality (Hedges’ *g* [95%CI] 1.3 [0.69–1.90]; *P* < 0.0001). Significant heterogeneity was found for Klotho (I^2^ = 90.69) responses. This is likely due to different conditions (healthy and diseased patients), protocols (aerobic, resistance, and aerobic + resistance training), and protocol durations in each study. Therefore, caution is required when interpreting the effect of exercise training on circulating Klotho values. We also performed the same meta-analysis with fixed-effect, and it did not deliver a different magnitude, effect, or significance compared with the random-effect meta-analysis.

### Sub-group analysis

**Figure 2:**
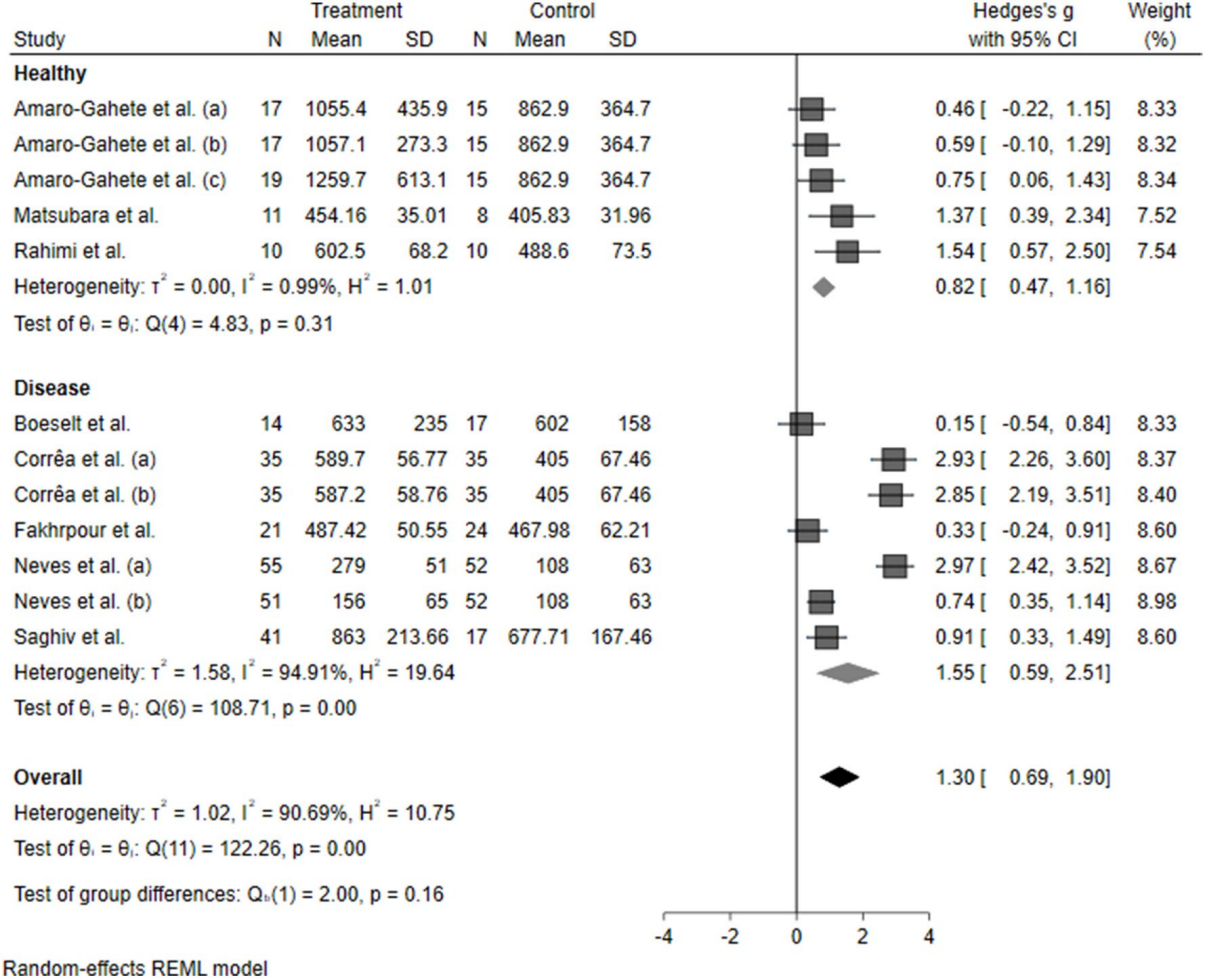
Forest plot of the results from a random-effects meta-analysis shown as Hedges’ g with 95% CIs of healthy and diseased patients on Klotho concentrations. For each study, the square represents the Hedges’ *g* of the intervention effect with the horizontal line intersecting it as the lower and upper limits of the 95% CI. The rhombi represent the weighted healthy, diseased, and total effect.

**Figure 3:**
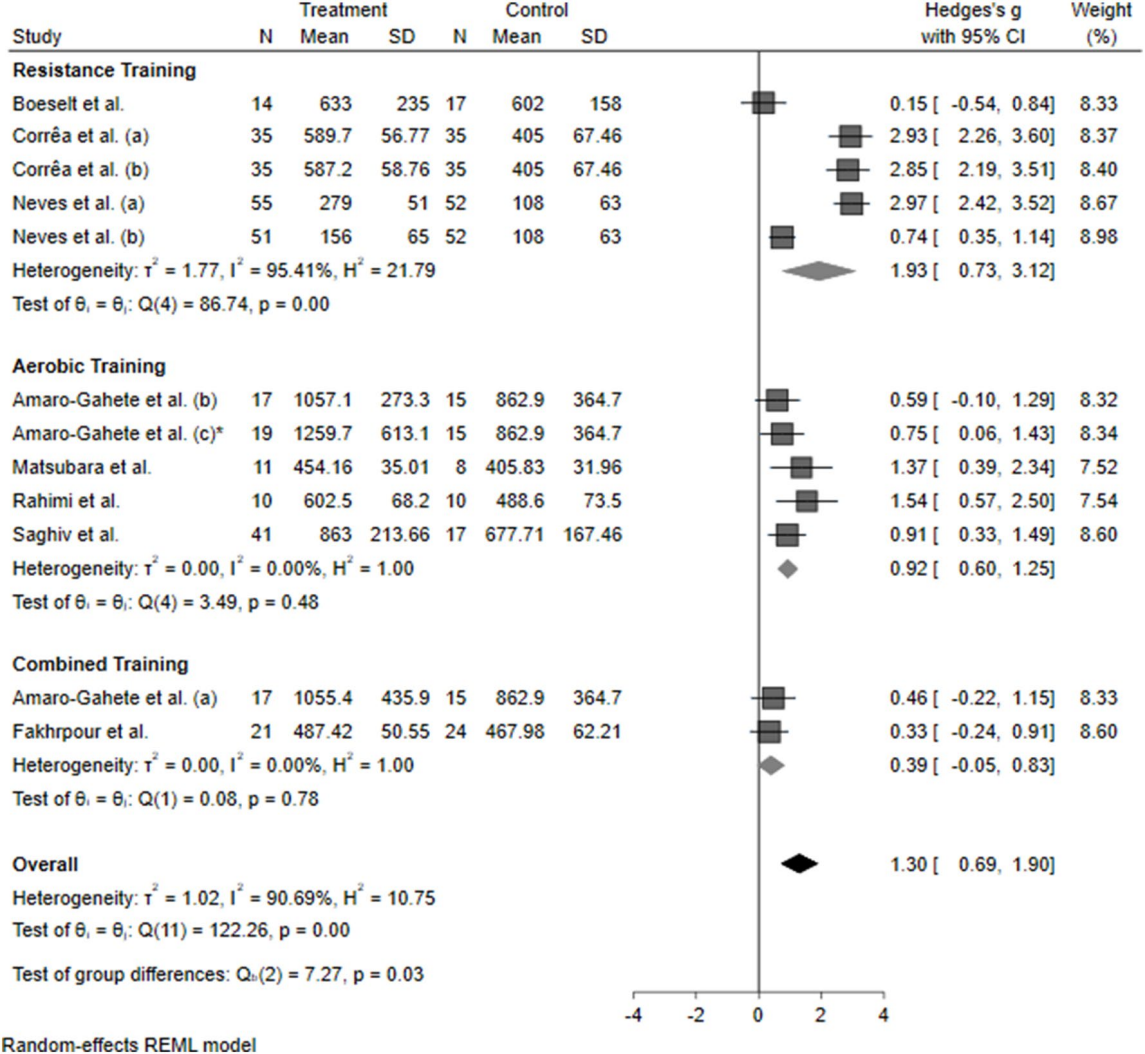
Forest plot of the results from a random-effects meta-analysis shown as Hedges’ g with 95% CIs of aerobic, resistance, and combined training on Klotho concentrations. For each study, the square represents the Hedges’ *g* of the intervention effect with the horizontal line intersecting it as the lower and upper limits of the 95% CI. The rhombi represent the weighted the aerobic, resistance, and combined training.

A sub-group meta-analysis was performed by stratifying the studies by population condition (healthy vs. presence of disease) and exercise training protocol (aerobic vs. resistance vs. combined training). The effects were maintained, independent of the condition (healthy: 0.82 [0.47–1.16], *P* < 0.0001); diseased: 1.55 [0.59–1.90], *P* < 0.0001), as described in Fig. 2. Klotho seems to increase significantly after resistance training (1.93 [0.73–3.12], *P* < 0.0001) and aerobic training (0.92 [0.60–1.25], *P* < 0.0001). However, there were no significant modifications in Klotho levels after combined training (0.39 [−0.05 to 0.83], *P* > 0.05), as described in Fig. 3. This response might be due to the different interventions, durations, and volumes, which could differentially influence the molecular pathways related to Klotho expression. Trying to address this issue, we performed a cumulative analysis of Klotho levels according to the protocol duration (12 to 24 weeks) and estimated training volume (60 to 210 min per week).

**Figure 4:**
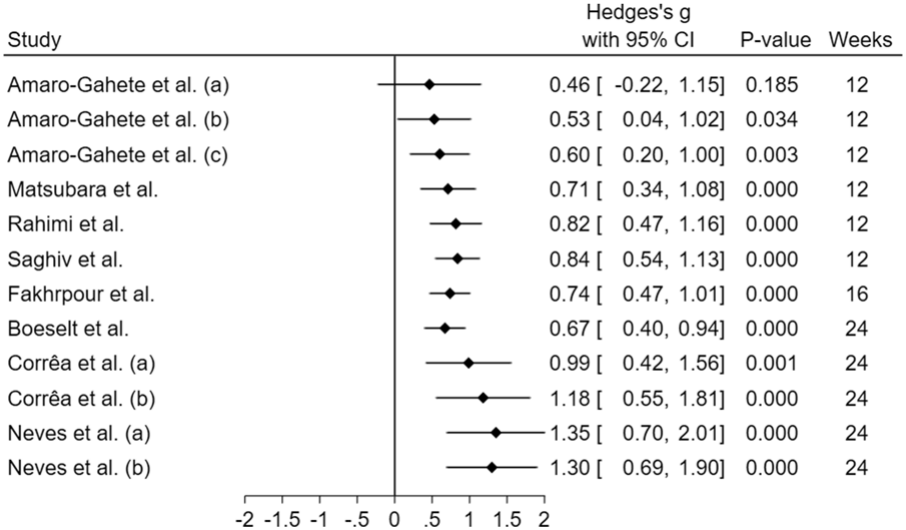
Cumulative forest plot from a random-effect meta-analysis shown as Hedges’ g with 95% CIs of the protocol duration increase on Klotho concentrations. For each study, the square represents the Hedges’ *g* of the intervention effect with the horizontal line intersecting it as the lower and upper limits of the 95% CI.

**Figure 5:**
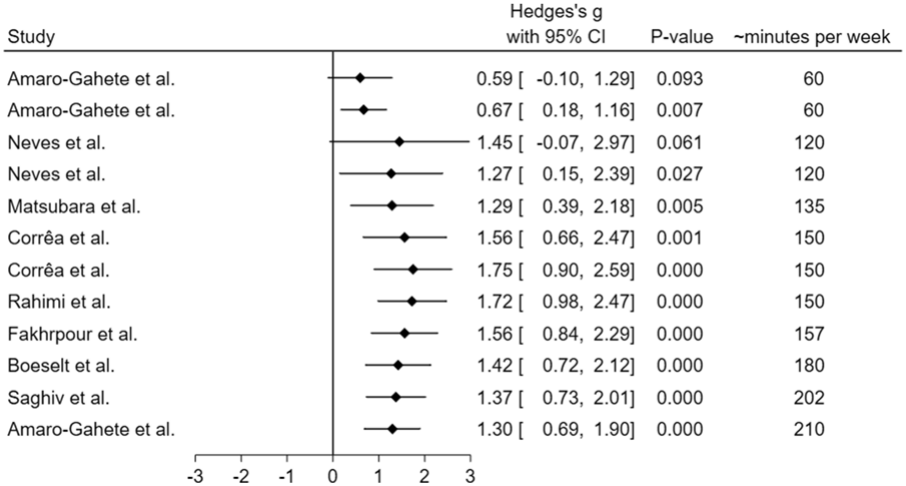
Cumulative forest plot from a random-effect meta-analysis shown as Hedges’ g with 95% CIs of the increase of minutes per week on Klotho concentrations. For each study, the square represents the Hedges’ *g* of the intervention effect with the horizontal line intersecting it as the lower and upper limits of the 95% CI.

Figure 4 illustrates the cumulative analysis of Klotho response according to protocol duration. Interestingly, the protocol’s length seems to play a key role in Klotho responses. Moreover, Fig. 5 illustrates a possible inverted “U” shaped curve, pointing to the possibility of a dose–response related to Klotho changes and the protocol volume (minutes per week) whereas ~ 150 min per week appears to present the highest magnitude of Klotho change. Therefore, we pooled together S-Klotho concentrations of the studies around^36,39,42^, below^35,37,41^, and above^35,38,40^ 150 min per week, and then analyzed them. The mean values were 1.65, 1.05, and 1.36, respectively, demonstrating a higher effect for the studies that performed a volume around 150 min per week.

### S-Klotho concentrations following exercise training in healthy and diseased subjects

Figure 3 of the Supplementary material shows that exercise training significantly increases Klotho in relation to the control group (S. Fig. 3A). This increase in mean values seems higher in healthy subjects (S. Fig. 3B,C).

## Discussion

To date, this is the first meta-analysis to assess the pooled effect of different exercise training protocols on blood S-Klotho levels. Our main finding was that exercise training consistently increases S-Klotho levels (Fig. 1). This response might occur without a relationship to the healthy status (Fig. 2) and training protocol (Fig. 3). There is an exception for combined training, which is probably related to the different protocol duration, intensity, or volume used in the studies that assessed the combined training. It is noteworthy that the findings regarding combined training should be interpretated with caution, since we found only two eligible studies.

A previous systematic review^43^ in both human and animal models demonstrated that exercise may increase S-Klotho levels. The authors also demonstrated Klotho as an important protein for hampering senescence. Although our meta-analysis was comprehensive and included multiple studies, some other studies that did not meet the inclusion criteria are also in agreement with our conclusions (Supplementary Table 1). In summary, the excluded studies demonstrated that master athletes present higher levels of S-Klotho in comparison to non-athletes^44,45^. This fact could be associated with physical activity levels, higher muscle mass, increased fat oxidation, low cardiometabolic risk, and muscle strength^46–52^. Moreover, a bout of acute exercise seems to increase S-Klotho in women^53^, and also in healthy football players^54^. However, military operational stress reduces Klotho in service members^55^. Taken together, it seems that an excessively stressful condition could blunt S-Klotho production.

### Exercise-induced S-Klotho: does oxidative stress play a role?

External and internal stress exposure plays a crucial role in exercise-induced molecules^56,57^. Strenuous exercise notably induces an increase in free radicals and reactive oxygen species, which is commonly known as oxidative stress^58^. This condition may lead to increase in muscle damage, toxins, and cell death^56,58,59^. In contrast, a transient increase in oxidative stress is necessary for aerobic-induced benefits, which normally trigger hormesis^56,59^. Hormesis is a common term used to describe an effect associated with toxic compounds that, in low doses, promote a beneficial effect on the exposed organism and, in high doses, present a toxic effect, leading to an inverse “U” shaped curve of optimal dose–response^60^. In this context, the relatively stable reactive oxygen species induced by muscle contraction, such as hydrogen peroxide and nitric oxide, may act as signaling molecules that improve cellular communication and function, aiming to reach stability^60^. Considering that S-Klotho may regulate and be regulated by reactive oxygen species, this protein increase should require a transient redox imbalance^61^. Another possible explanation on the exercise-induced Klotho levels might be related to the anti-inflammatory role of exercise. It known that inflammation decreases Klotho expression, leading to pre-mature aging and age-related issues^62,63^. Therefore, it is worthy to state that exercise-controlled inflammation may play a key role in increasing Klotho levels in human.

In our study, S-Klotho appears to be higher in trained people, regardless of the volume of training. Nevertheless, ~ 150 min per week seems to be the optimal volume to induce S-Klotho changes. This might be due to the transient increase in oxidative stress, leading to an upregulation of the antioxidant system and anti-inflammatory profile^64–68^. Considering that high exercise intensities might cause renal artery vasoconstriction^69^, the hypoxia induced by a limited blood-flow may promote a transient oxidative stress that would increase the antioxidant system, including S-Klotho. Taken together, there might be an optimal stress level for Klotho induction, and further studies should investigate the dose–response kinetics of this protein following different intensities and volumes. Finding the dose–response of exercise for this protein could lead to a better protocol prescription aimed at anti-aging effects, or assist in the assembly of training programs and periodization in high-performance sport. In summary, S-Klotho may be one of the main parts of a myriad of molecules that allow exercise training to be effective in the prevention and treatment of a plethora of diseases. Nevertheless, further studies should test this hypothesis.

### Exercise training protocols in S-Klotho response

Exercise training seems to stimulate S-Klotho regardless of the protocol (resistance or aerobic training). However, even subgrouping the meta-analysis according to the type of intervention, we could observe some divergences in both resistance and aerobic training. From the studies with resistance training^36–38^, all of them applied full-body resistance training, using exercises that require several muscle groupings in the same movement. Boeselt et al.^38^ applied a total of four exercises, Neves et al.^37^ used a total of twelve exercises (for both dynamic and isometric training), and Corrêa et al.^36^ used eight exercises (for both training with and without blood-flow restriction).

As observed in Fig. 3, the study of Boeselt et al.^38^ did not present a significant effect that favors intervention. The protocol consisted of 15–20 sets of 2–4 repetitions. Although their final workload was similar to the other included studies, it is known that different training configurations, such as number of sets and repetitions, produce distinct metabolic responses in humans^70^. Moreover, time under tension is another variable that may influence the responses following resistance training^71,72^. Burd et al.^71^ provided evidence that greater muscle time under tension could increase mitochondrial and sarcoplasmic protein synthesis. Considering that Klotho is mainly influenced by metabolism^11,14,20^, it is possible that different resistance exercise prescriptions may lead to different S-Klotho responses.

Regarding the studies that applied aerobic training, two studies used a treadmill for the intervention^35,40^, one study used a cycle-ergometer^41^, and one study performed aerobic aquatic training^42^. There are plenty of physiological differences between these interventions^73,74^. However, all the included studies that applied an aerobic training protocol presented similar responses in S-Klotho production. This might be due to the impact of aerobic exercise stimulation on mitochondrial biogenesis^75^. As stated before, oxidative metabolism may influence Klotho production. Thus, aerobic training probably induces S-Klotho regardless of the protocol configuration, due to the overall mitochondrial stimulus.

Considering the aforementioned points, a combination of aerobic + resistance training should be the optimal protocol to increase S-Klotho in humans. To our surprise, there was no significant effect of this training intervention on Klotho. However, the present data should be interpreted with caution, because only two studies investigated the combined effect of both training models^35,39^. Moreover, we should consider all training configurations in the process to change molecular signatures in the organism (intensity and volume). In this regard, we hereby suggest a possible role of the training-volume-dependent manner to different concentrations of S-Klotho in humans.

### Klotho: a potent target for exercise mimetics

We must highlight that an exercise mimetic is probably not the best intervention and will not solve physical inactivity, as already stated elsewhere^32^. Nonetheless, the potential role of an “exercise pill” is to promote benefits for populations that cannot perform physical activity. In the 30-year follow-up to the Dallas bedrest study^76^, 3 weeks of bedrest in 20-year-old men had a more profound impact on cardiovascular parameters than 30 years of aging. Considering that a person with spinal cord injury, coma, or post-surgical stage may spend up to 3 weeks in bedrest, a key question is raised here: can exercise mimetics mitigate the impacts caused by these conditions?

Here, we demonstrated Klotho as an emerging exerkine induced by different protocols of exercise training, providing clues to future investigations that might consider this anti-aging protein as a potent bioactive compound for the development of exercise mimetics.

### Klotho: a potent target for sports sciences and medicine

The prevention and rehabilitation of various sports injuries has been widely studied^77–80^. Baseline characteristics such as muscle strength, body composition, and levels of athleticism may influence injury prediction^78^. Such properties seem to be constantly modulated by several molecule pathways, which include Klotho^81–83^. In rodents, a reduction in this protein led to the loss of muscle stem cell function^81^ and appeared to modulate myogenesis-accelerated muscle growth after injury^83^. In addition, a decrease in Klotho expression can contribute to cartilage damage in osteoarthritic mice^82^. Taken together, all this evidence points to a possible role of Klotho in the management of muscle and cartilage injuries.

Although most of the included studies demonstrated that chronic exercise training increases S-Klotho levels in humans, none of them presents a causal effect between Klotho and exercise-induced beneficial outcomes, such as functional performance and body composition. However, Phelps et al. showed that Klotho expression is required to enable exercise to have an effect on endurance capacity and skeletal muscle strength^84^. Sahu et al. verified that knocking down the Klotho gene in vivo appears to hamper the progression of muscle progenitor cell lineage, blunting muscle fiber regeneration^85^. This gene knockdown also provoked damage to mitochondrial DNA and impaired cellular bioenergetics. Therefore, the increase in S-Klotho identified in the present study might point towards a possible biomarker of the training status and it should be targeted by future studies, aiming to verify the causality between Klotho and the benefits outlined in this study induced by exercise training in humans.

## Conclusion and outlook

In summary, our findings add new information about the effects of different exercise training protocols on S-Klotho levels. Another key message of the present study is that although the overall results support the claim that Klotho might be an exerkine associated with a myriad of health benefits, there is no consensus on the ideal exercise protocol to lead to a greater increase in Klotho concentrations. Furthermore, we speculate that Klotho may act as an exerkine, and its kinetics could be modified according to training volume, intensity, and duration. Klotho can possibly be used alone or in conjunction with other exerkines and baseline characteristics, through dynamic mathematical modeling, to assist physiologists and coaches in high-performance sports. Finally, Klotho is a candidate that should be targeted and explored by industries and researchers aiming to build and develop an exercise mimetic biotool. However, this conclusion should be interpretated with caution since the population and the protocol appear indicating possible sources of heterogeneity and the lower number of studies and samples.

## Methods

### Protocols and registration

We first performed a systematic review according to the Cochrane Handbook recommendations^86^ and reported the results according to the Preferred Reporting Items for Systematic Reviews and Meta-analyses (PRISMA) guidelines^87^. The protocol for this review was registered in the International Prospective Register of Systematic Reviews https://www.crd.york.ac.uk/prospero/display_record.php?ID=CRD42021243080 with registration number: CRD42021243080.

### Criteria for considering studies for this review

We included randomized and quasi-randomized controlled trials (RCTs) that investigated the S-Klotho response after the following comparisons: (a) Resistance training versus no training; (b) aerobic training versus no training; (c) combined resistance + aerobic training versus no training. To be included in this review, studies needed to apply a chronic exercise protocol in humans.

No language restriction was applied in the search. There were no limitations on age, sex, or condition (diseased or healthy) since Klotho could be induced by exercise in any populations.

### Search strategy and selection criteria

Two authors (TA and AR) independently reviewed published studies by searching the Cochrane Central Register of Controlled Trials (CENTRAL), MEDLINE through PubMed, EMBASE, CINAHL, CT.gov, and PEDro from their inception through March 2021. All searches were adapted from the MEDLINE search strategy as reported: ("Exercise training” [MeSH]) AND ("klotho proteins" [MeSH). We reviewed the trials’ bibliographies, identifying and contacting some of the authors in the field to clarify trial eligibility or to identify additional published or unpublished data. Noteworthy, unpublished data was sought according to Young and Hopewell^88^.

### Selection of the studies

Two review authors (TA and AR) independently checked the references identified by the search strategy. The full texts of all potentially relevant studies were obtained for independent assessment. Disagreements were solved through discussion, and a third review author (HLC) acted as arbitrator where necessary. All citations were downloaded into EndNote X9^®^, duplicates were removed, and an identification number was assigned to each article.

### Data extraction

The same authors collected data in sufficient detail in order to better extract properties including studies based on PICO: Population: humans; Intervention: exercise training; Comparator, no exercise group; Outcome: Klotho response. We also extracted the associated benefits related to the Klotho response in each study. After extracting the data, two authors (TA and AR) graded the risk of bias in the included trials. Disagreements were resolved through discussion and a third reviewer (HLC) acted as moderator where necessary. Authors of primary studies did not extract data from their own studies. AR entered the data into the Software ReviewManager 5.4. (RevMan 5.4.) and HLC checked data entry. To plot results in graphs, WebPlotDigitizer v.4.1 (Austin, Texas, USA) software was used.

### Assessment of risk of bias in the included studies

Two review authors independently assessed the risk of bias of all included studies. The assessment was according to the Cochrane Handbook for Systematic Reviews of Interventions^86^. The included studies were evaluated according to randomization sequence generation, allocation concealment, blinding (participants and outcomes), incomplete outcome data, selective outcome reporting, and other sources of bias. Disagreements were resolved by discussion between the two authors. The risk of bias was graded as “high risk of bias”, low risk of bias”, or “unclear risk of bias”.

### Measures of exercise training effect and heterogeneity

Owing to the anticipated heterogeneity across studies due to different populations and conditions (health and diseases), we conducted a meta-analysis of random effects. The meta-analysis was performed with STATA 16 (StataCorp. 2019. *Stata Statistical Software: Release 16*. College Station, TX: StataCorp LLC). The same analyses were also performed in RevMan 5 to check the agreement between them and avoid errors. Due to the variety of kits used by the included studies to assess Klotho concentration, we used the standardized mean difference with corresponding 95% confidence intervals (CI) to calculate Hedges’ (adjusted) *g*. We took data from the post-training and post-control period. Studies with multiple treatment groups were split according to the type of intervention and analyzed as an independent study. Heterogeneity was assessed by visual inspection of the forest plots and by the I^2^ statistics^86^. As recommended, the I^2^ was interpreted as follows: 0–40% might not be important heterogeneity; 30–60% moderate heterogeneity; 50–90% substantial heterogeneity. P < 0.10 was adopted to point out statistically significant heterogeneity. The potential reasons for the heterogeneity were assessed through subgroup analysis.

### Subgroup analysis

The subgroup analysis was performed with the variables that may influence the expression and production of molecular substances induced by exercise training. Therefore, one condition was the population: healthy vs. diseased patients. Another condition was the protocol of exercise training: aerobic vs. resistance vs. combined training. We also performed a cumulative analysis of the protocol duration (12 to 24 weeks) and the estimated volume (60 to 210 min per week) adopted in each study to verify the Klotho kinetics according to time.

### Additional analysis

The mean values and deltas (post–pre) were plotted on GraphPad Prism version 8.0.0 for Windows (GraphPad Software, San Diego, California USA). A student-t test was performed comparing the deltas of Klotho concentrations in control group *vs.* exercise group. A three-way analysis of variance 2 × 2 × 2 (Intervention × Time × Condition) was performed to compare Klotho concentrations among healthy and diseased patients. A two-way mixed analysis of variance 2 × 2 (Intervention x Condition) was applied to compare the deltas of Klotho levels among groups. *P* < 0.05 was adopted for statistical significance. Furthermore, we constructed a table summarizing the main characteristics of the excluded studies (Supplementary Table 1).

## Supplementary Information

Supplementary Information.

Supplementary Video 1.
