# Supplementary material for: A systematic review and meta-analysis demonstrating Klotho as an emerging exerkine
Source: Sci Rep. 2022 Oct 20;12:17587. doi: 10.1038/s41598-022-22123-1 (PMC9585050; doi:10.1038/s41598-022-22123-1)
Supplement: Supplementary file 1 — Supplementary Information. [file 41598_2022_22123_MOESM1_ESM.docx]

**Supplementary Figure 1 -**PRISMA flow diagram

**Identification of studies via other methods**

**Identification of studies via databases and registers**

Records identified from:

Websites (n = 3)

Citation searching (n = 3)

Records removed *before screening*:

Duplicate records removed (n = 50)

Records removed for other reasons (n = 1)

Records identified from databases:

Total (n = 153)

**Identification**

Records screened

(n = 102)

Records excluded

(n = 62)

Reports not retrieved

(n = 1)

Reports sought for retrieval

(n = 6)

Reports sought for retrieval

(n = 20)

Reports not retrieved

(n = 10)

**Screening**

Reports assessed for eligibility

(n = 5)

Reports excluded:

Cross-over (n =1)

Animal model (n = 1)

Reports assessed for eligibility

(n = 50)

Reports excluded:

Observational study (n = 7)

Acute intervention (n = 3)

Animal model (n = 29)

Not met the inclusion criteria (n=6)

Studies included in review

(n = 5)

Reports of new included studies

(n = 3)

**Included**

**Supplementary figure 2.** Risk of bias of the included studies


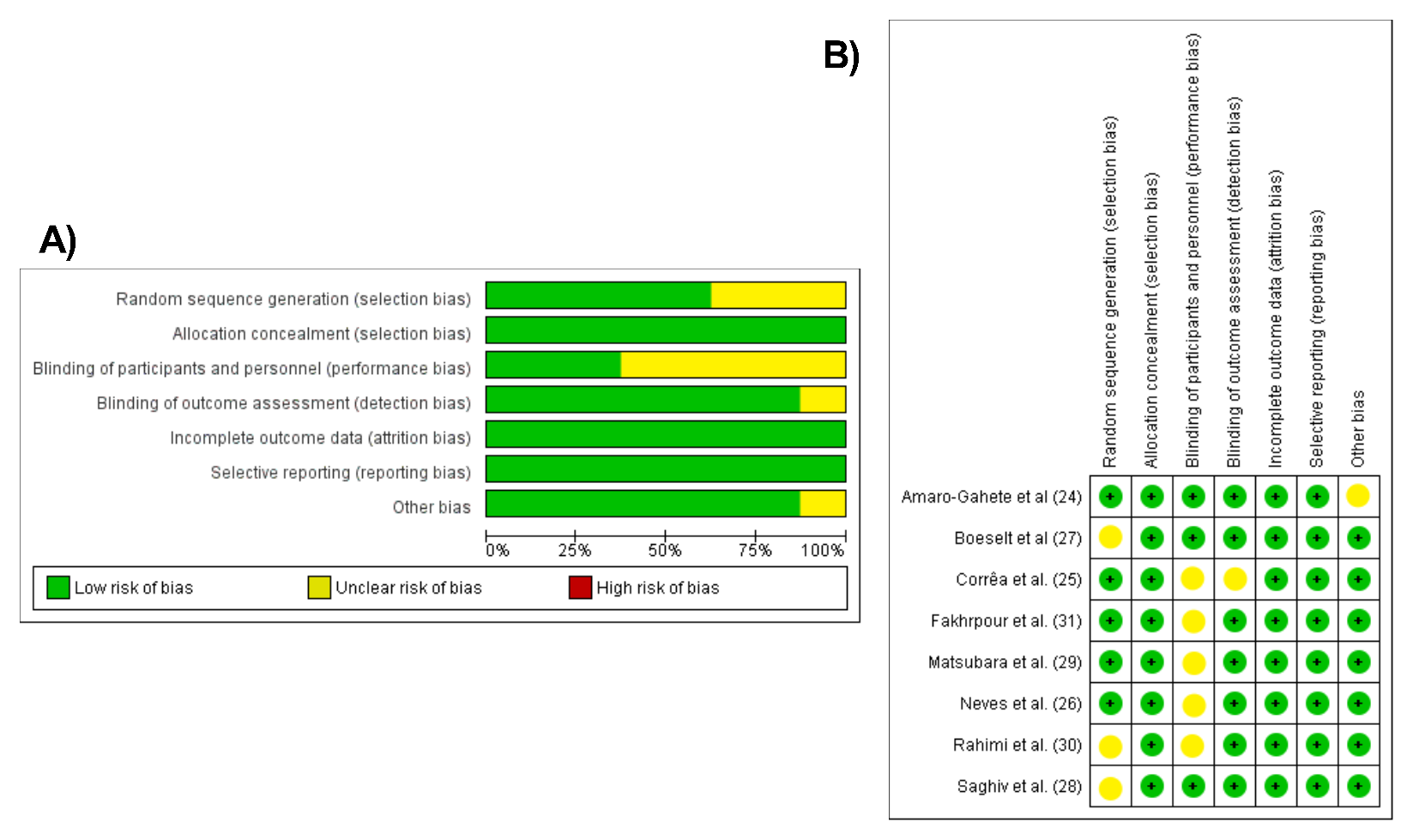


**Supplementary figure 3.** Additional analysis. Delta difference of the mean values of S-Klotho in control and exercise trained groups (A). Mean values of Klotho according to the condition (healthy and disease) (B). Delta of Klotho concentrations in control and exercise groups according to the condition (C); a *P* < 0.05 *vs.* Control groups; b *P* < 0.05 *vs.* healthy exercise trained groups

**
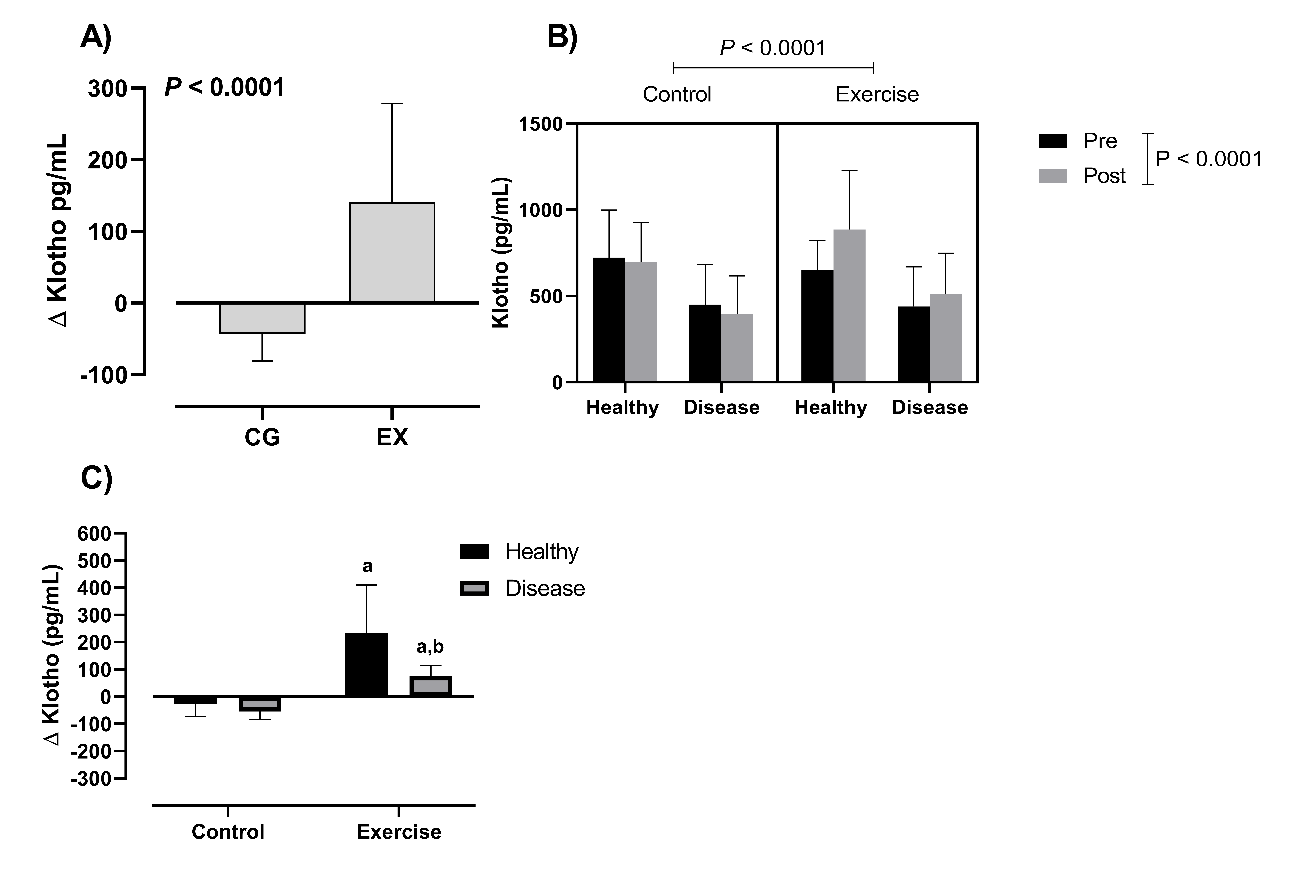
**

**Supplementary Table 1.** Characteristics of the excluded studies

| **Supplementary Table 1-** Characteristics of the studies that had their full-text read but was excluded. | | | | |
| --- | --- | --- | --- | --- |
| Study | Population | Condition | Outcome | Type of the study |
| Amaro-Gahete et al. (35) | Sedentary middle-aged adults | Healthy | Klotho associated with fat oxidation | Cross-sectional |
| Amaro-Gahete et al. (41) | Sedentary middle-aged adults | Healthy | Klotho associated with physical activity levels | Cross-sectional |
| Amaro-Gahete et al. (36) | Sedentary middle-aged adults | Healthy | Klotho associated with better body composition | Cross-sectional |
| Amaro-Gahete et al. (37) | Sedentary middle-aged adults | Healthy | Klotho associated with lower cardiometabolic risk | Cross-sectional |
| Beckner et al. (44) | Service members | Simulated military operational stress | Military operational stress reduced Klotho from baseline | Acute |
| Santos-Dias et al. (42) | Men and women | Healthy | Exercise induced klotho levels in women | Acute |
| Semba et al. (38) | Older community dwelling | Healthy | Lower levels of klotho are associated with poor muscle strength | Longitudinal study |
| Castro et al. (39) | Older community dwelling | Healthy | Klotho levels are associated with activities of daily living | Longitudinal study |
| Shardell et al. (40) | Older community dwelling | Healthy | Klotho levels are associated with lower-extremity physical performance | Longitudinal study |
| Mostafidi et al. (43) | Football players | Healthy | Aerobic exercise increased Klotho levels | Acute |
| Rosa et al. (33) | Athletes | Master athletes | Master athletes presented higher levels of Klotho in relation to middle-aged control | Cross-sectional |
| Rosa et al. (34) | Athletes | Master athletes | Sprinters presents higher levels of Klotho comparing to endurance athletes | Cross-sectional |
